# An Optimized Most Probable Number (MPN) Method to Assess the Number of Thermophilic Free-Living Amoebae (FLA) in Water Samples

**DOI:** 10.3390/pathogens9050409

**Published:** 2020-05-24

**Authors:** Mirna Moussa, Isabel Marcelino, Vincent Richard, Jérôme Guerlotté, Antoine Talarmin

**Affiliations:** 1 Unité TReD-Path (Transmission Réservoir & Diversité des Pathogènes), Institut Pasteur de la Guadeloupe, Les Abymes, 97183 Guadeloupe, France; mirna.moussa.83@gmail.com (M.M.); ATALARMIN@pasteur-guadeloupe.fr (A.T.); 2 Institut Pasteur, 75015 Paris, France; vincent.richard@pasteur.fr; 3 Institut de Systématique, Evolution, Biodiversité (ISYEB) MNHN, CNRS, Sorbonne Université, EPHE Université des Antilles, Pointe-à-Pitre, 97110 Guadeloupe, France; jerome.guerlotte@univ-antilles.fr

**Keywords:** free-living amoebae, thermophilic amoebae, *Naegleria fowleri*, most probable number (MPN), optimized quantification method

## Abstract

Detection and quantification of pathogenic free-living amoebae (FLA) in water samples is critical for assessing water quality and for disease management issues. The most probable number (MPN) is commonly used to account for FLA in water. Nevertheless, this requires a high number of water replicates and working volumes, and a consequent number of non-nutrient agar (NNA)-plates seeded with *Escherichia coli.* Herein, we aimed at optimizing this difficult method, taking also into account key factors such as (i) the counting method, (ii) the delay between sample collection and sample processing, and (iii) the temperature during water sample transportation. To simplify the MPN method, we filtrated 1 × 1000 and 1 × 100 mL water samples, and cellulose acetate filters were cut in 10 parts and inverted on NNA-plates overlaid with *E. coli*. The comparison between the classical and our optimized MPN method showed that the final counts were similar, therefore validating the use of the optimized method. Our results also showed that for thermophilic FLA (such as *Naegleria fowleri*), water samples can be kept at around +30°C and processed within 24 h. This improved MPN method is now routinely used in our laboratory to control *Naegleria* sp. in the water samples in Guadeloupe.

## 1. Introduction

Free-living amoebae (FLA) are ubiquitous unicellular organisms, being found in water, soil, dust, and air samples. Some FLA are thermophilic, being able to survive and/or replicate at temperatures equal or above 37 °C [1,2,3]. Amongst these, some can be pathogenic to humans and animals such as *Acanthamoeba* sp., *Naegleria* sp., and *Balamuthia mandrillaris* [4,5]. Others are nonpathogenic but of medical importance because they can act as hosts, vehicles, and training grounds for bacteria, such as *Vannella* sp. [6] and *Vermamoeba vermiformis* [7,8,9,10]. 

The thermophilic FLA *Naegleria fowleri* is frequently found in freshwater and soil [11,12], and it may cause primary amoebic meningoencephalitis (PAM), a rare but often fatal disease of the central nervous system [13]. It is generally acquired while swimming or during other recreational activities in freshwater lakes and ponds. Infection of the brain occurs after *N. fowleri* reaches the nasal cavity and invades the nasal mucosa. Afterwards, *N. fowleri* penetrates the nasal epithelium to the olfactory nerves and migrates through the cribriform plate to invade the brain and meninges [14]. 

In Guadeloupe (French West Indies), a fatal case of PAM occurred in 2008 after a child swam in a bath fed with geothermal waters [15]. In 2013, our group revealed that thermophilic FLA (and in particular, *N. fowleri*) were frequently detected in these baths [12]; the contamination of the water with *N. fowleri* occurs after emerging from the geothermal source, when the water runs over the soil [11]. We also observed that *N. fowleri* was often found at low concentrations, below 22 amoebae per liter [12]. These low concentrations in environmental water often make it difficult to accurately count the FLA [16], regardless of the method that is used to concentrate the samples, either by centrifugation or filtration [17]. Because of the risk associated to the presence of *N. fowleri* (the presence of one single amoeba is enough to cause or start an infection) and the potential link between the concentration of *N. fowleri* and pathogenicity, it is important to obtain the most accurate count of this amoeba in water [18,19,20].

Several methods have been developed to detect and enumerate thermophilic and pathogenic FLA, such as loop-mediated isothermal amplification (LAMP) [21], PCR (conventional and quantitative), and cytometry [16,17,22,23,24,25]. Still, the most probable number (MPN) method (followed by FLA identification by microscopic observation and/or PCR) is the most commonly used to estimate the concentration of viable microorganisms in a sample by means of replicate amoeba growth in ten-fold dilutions [26,27,28,29]. Nevertheless, this is time-consuming and requires a high number of water sample replicates and working volumes and a consequent number of non-nutrient agar (NNA)-plates seeded with *E. coli*.

Herein, we aimed at optimizing this MPN method by reducing the working volumes and number of replicates. Additionally, we evaluated the impact of the delay between sample collection and sample processing, and the temperature during water sample transportation, on the counts of thermophilic FLA.

## 2. Results

### 2.1. Optimization of the MPN Method

In Table 1, we present the number of thermophilic FLA (sensu lato) and, in particular, for *N. fowleri* obtained after using the classical (cMPN) and the modified (mMPN) MPN methods. The results show that for FLA, the percentage of samples with equal or higher counts obtained with the mMPN method compared to those obtained with the classic method are 84% (16/19 samples) for both 100 mL boxes and 10 mL boxes. Nevertheless, regardless of the sample, the slight differences are not statistically significant (Mc Nemar Test, *p*-value = 0.48 for 100 mL boxes and *p*-value = 0.70 for 10 mL boxes). Moreover, only 2 samples (samples 1 and 13) tested positive for *N. fowleri* using the cMPN, while the optimized method revealed the presence of this amoeba in six water samples (samples 1, 8, 13, 14, 15, and 19).

As we were concerned about accurately assessing the count of pathogenic *N. fowleri*, we also seeded water samples with known quantities of pure *N. fowleri*. For this, we counted the number of positive dilutions (for cMPN) or the number of positive filters (for mMPN) for amoeba growth and reported these values to MPN tables [30]. Indeed, using a 100 mL sample volume, we obtained a higher number of *N. fowleri* colonies with the mMPN compared to the cMPN (*p* = 0.03, Wilcoxon test), although the number of amoebae/L artificially seeded was within the range of counts obtained with both MPN methods. 

### 2.2. Assessment of the Time and Storage Temperature of Water Samples, Prior to Processing

**Figure 1:**
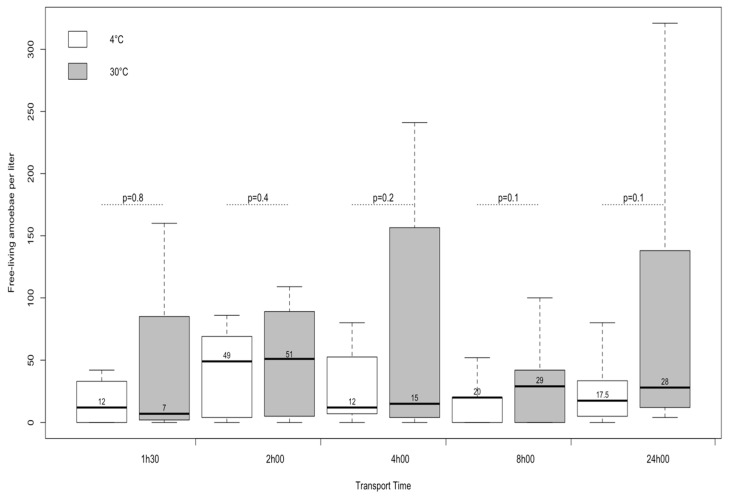
Median number of thermophilic amoebae per liter of water, at different times post-water sampling and at two storage temperatures (+4 °C or +30 °C) (box 1° and 3° quartile).

To study the effect of temperature on FLA prior to water sample processing, we selected two temperatures: 4 °C (usually used for samples storage) [26] and 30 °C, the average daily maximum air temperature in Guadeloupe. The results presented in Figure 1 show that the median numbers of thermophilic amoebae were not significantly different according to the temperature. 

Regarding the time before sample processing, we selected several time windows ranging from 90 min to 2 h [26], 4 and 8 h (which is the maximum if the samples have to be processed on the same day as the sampling), and up to 24 h (in case water samples come from neighboring islands). Our results showed that there was no significant difference in the median number of thermophilic amoebae regardless of the delay between the collection and the culture. 

## 3. Discussion

Thermophilic FLA, and in particular *Naegleria* sp., have been frequently detected in Guadeloupe, in soil [11] and in natural thermal water [12]. French health authorities have endorsed a rule that forbids swimming or playing sports in natural water where the concentration of *N. fowleri* is above 100 amoebae/L [18]. As a preventive health measure, the local French health agency (ARS Guadeloupe) requests that we investigate 3–4 times per year the presence of *Naegleria* sp. (in particular *N. fowleri*) in the geothermal recreational waters often frequented by tourists and Guadeloupians. It is thus important to have an easy-to-use and reliable method to account for these FLA. 

Many rapid detection assays have recently been developed and optimized to overcome conventional culture and microscopy techniques to detect *N*. *fowleri* in clinical and environmental samples. Many other studies also reported the superior sensitivity of real-time PCR over PCR, immunohistochemistry, or culture [16,17,21,22,23,24,25]. Herein, we used the MPN method because it allows identifying the number of viable, thermophilic, and cultivable FLA, such as *Acanthamoeba*, *Naegleria, Vannella*, and *Vermamoeba*. FLA viability can reflect the ability of trophozoites to multiply in water and the high resistance of FLA cysts to water treatments. Therefore, we can determine the potential dissemination of pathogenic FLA amoebae in habitats related to human population.

However, it is widely known that the classical MPN method (using either filtration or centrifugation to recover the FLA) is time-consuming, even if only performed with five replicates [27]. Our work clearly shows that this method can be substantially simplified. Indeed, our method using two samples (1000 and 100 mL) filtered once is cheaper and much simpler than other methods, but most importantly, it is also reliable. Although we used a small number of water samples in our study, the new method gives results (FLA/L) similar to those obtained with the cMPN and enabled accounting for equal or more *N. fowleri* than the cMPN method. We believe that cutting a filter in 10 equal pieces instead of two increases the probability of amoebae getting in contact with *E. coli* seeded on the NNA, promoting their growth, and therefore reduces the competition between *N. fowleri* and other FLA for food [2]. This is particularly important when it comes to having a good estimate of *N. fowleri* concentrations in water. 

This optimized method has an additional advantage: ease of sample transportation. With the mMPN method, we only need two sterile flasks per site, which can be transported at ambient temperature and can be processed within 24 h post-collection. These are very important features to take into account when it is necessary to perform water sampling in hot springs that are remote and difficult to access by car. Since natural thermal water baths are of small size in Guadeloupe (from 8 to 40 m^2^), this sampling is enough to give an indication of the contamination by *N. fowleri.*

Globally, our study demonstrates that this optimized method can replace the classical MPN method in any laboratory working on FLA. For instance, it is now routinely used in our laboratory to control *Naegleria* sp. in the water samples in Guadeloupe. 

## 4. Materials and Methods 

### 4.1. Water Samples and Amoeba Isolation

Two types of water samples were used: (i) those collected from 19 geothermal baths in Guadeloupe and (ii) five which were prepared with natural geothermal water that was filtered and then seeded with *N. fowleri* (previously isolated by our team from a hot spring in Guadeloupe) at different concentrations ranging from 2 to 36 *N. fowleri* per liter (Table 1). All samples were pressure-filtered through a cellulose nitrate filter (pore size, 1.2 μm; diameter, 47 mm, Millipore (FisherScientific)) [16,31].

### 4.2. The Most Probable Number (MPN) Methods

To compare the two counting methods, the water samples mentioned above were divided into two equal parts: one to be tested by the classical MPN method (cMPN) and the other with the modified MPN method (mMPN). The two methods are described below. All the plates for both procedures were incubated at 44 °C.

#### 4.2.1. Classical MPN Method

For this, water samples of 1 L and 100 mL were filtered as follows: 10 × 100 and 10 × 10 mL, respectively [17,26,27]. Each filter was cut into two pieces, and placed inverted on a 1.5 % non-nutrient agar plate seeded with a thin layer of *Escherichia coli* (NNA–*E. coli*), i.e., two pieces of filter per plate, per volume [17].

#### 4.2.2. Optimized MPN Method

When 1 L and 100 mL water samples were collected, each sample was filtered only one time for each volume. Each filter was cut into 10 equal pieces (passing through the center of the filter), and placed inverted on a 1.5 % NNA–*E. coli* plate, i.e., ten pieces of filter per plate, per volume.

### 4.3. Amoeba Identification and Counting

During incubation, the first-line plates were examined daily macroscopically and microscopically using an inverted microscope. This allowed us to see the development of lytic areas over the bacterial coating, which corresponds to amoebic growth. Every amoeba plaque emerging along the filters was picked and subcultured on fresh NNA–*E. coli* (second-line plates). The isolates were pre-identified morphologically using Page’s taxonomy key [32]. Amoeba identification was then performed by PCR using ITS and NFITS primers, as previously described [12,33].

For the cMPN method, the concentration of thermophilic FLA and *N. fowleri* was obtained by counting the total number of positive plates or each volume and by referring to the MPN table [34].

For the mMPN, the number of positive filter pieces was counted. Each filter piece obtained after filtering 100 mL was considered to be 10 mL of water, and each filter piece obtained after filtering 1 L was considered to be 100 mL of water. The numbers of positive piece of filter for each volume were reported in the same MPN statistical table.

### 4.4. Assessment of Storage Temperature and Delay of Delivery of Water Samples 

To evaluate the impact of temperature storage and time on amoeba recovery, before filtration, we used eight water samples. Each sample was separated in 10 parts: Five were kept at ambient temperature (+30 °C), and the others were stored at +4 °C. At different times post-collection, one sample that was stored at ambient temperature and one that was stored at +4 °C were filtered and cultured. For this, we used the classical MPN method by filtering 1100 mL of water as described above. 

### 4.5. Statistical Analyses 

All statistical analyses were performed using R software, version 3.5.2 (R Foundation for Statistical Computing, Vienna, Austria. URL https://www.R-project.org/). The means of positives cultures and the estimated numbers of colonies obtained after new vs. classical MPN methods, and mean numbers of colonies obtained after transportation at +4 vs. +30 °C were compared using the nonparametric Wilcoxon rank test for paired series. The mean numbers of colonies that were obtained with a transportation delay were compared using the Kruskal–Wallis nonparametric test. For all comparisons, *p*-values < 0.05 were considered to be statistically significant.

## Figures and Tables

**Table 1:** The number of thermophilic free-living amoeba and *Naegleria fowleri* colonies observed and the most probable number obtained with the classical most probable number (MPN) method and the modified MPN method.

Thermophilic FLA							*Naegleria fowleri*					
Samples	Classical MPN Method			Modified MPN Method			Classical MPN Method			Modified MPN Method		
	Positive Petri Boxes		MPN Number (FLA/L)	Positive Filter Pieces		MPN Number (FLA /L)	Positive Petri Boxes		MPN Number *Nf*/L	Positive Filter Pieces		MPN Number *Nf*/L
	100 mL	10 mL		100 mL	10 mL		100 mL	10 mL		100 mL	10 mL	
1	10	3	86 (39–191)	9	6	80 (37–175)	3	0	7 (3–20)	1	2	6 (2–19)
2	3	3	14 (6–31)	5	3	21 (10–43)	0	0	<2	0	0	<2
3	10	10	>461	10	10	>461	0	0	<2	0	0	<2
4	1	0	2 (1–14)	1	0	2 (1–14)	0	0	<2	0	0	<2
5	0	0	<2	0	0	<2	0	0	<2	0	0	<2
6	10	10	>461	10	10	>461	0	0	<2	0	0	<2
7	10	8	321(150–696)	10	7	241 (110–529)	0	0	<2	0	0	<2
8	1	0	2 (1–14)	2	0	5 (1–16)	0	0	<2	1	0	2 (1–14)
9	0	0	<2	0	0	<2	0	0	<2	0	0	<2
10	1	0	2 (1–14)	1	0	2 (1–14)	0	0	<2	0	0	<2
11	3	0	7 (3–20)	0	0	<2	0	0	<2	0	0	<2
12	7	0	21(10–43)	4	1	12(5–28)	0	0	<2	0	0	<2
13	7	0	21(10–43)	7	4	35 (18–70)	1	0	2 (1–14)	1	0	2 (1–14)
14	10	7	241 (110–529)	10	10	>461	0	0	<2	1	1	4 (1–16)
15	4	0	10(4–25)	7	0	13 (6–30)	0	0	<2	1	0	2 (1–14)
16	2	0	5 (1–16)	5	0	13 (6–30)	0	0	<2	0	0	<2
17	0	0	<2	0	0	<2	0	0	<2	0	0	<2
18	4	1	12 (5–28)	5	0	13 (6–30)	0	0	<2	0	0	<2
19	10	10	>461	10	4	109 (47–253)	0	0	<2	1	0	2 (1–14)
20*							8	0	26 (13–54)	9	0	35 (18–69)
21*							8	0	26 (13–54)	9	0	35 (18–69)
22*							4	1	12 (5–28)	6	0	16 (8–36)
23*							3	0	7 (3–20)	4	0	10 (4–25)
24*							1	0	2 (1–14)	2	0	5 (1–16)

FLA/L: Number of free-living amoebae per liter. ***Nf*/L:** Number of *Naegleria fowleri* per liter. *20, 21, 22, 23, 24: Water doped with 36, 18, 8, 4, and 2 *Naegleria fowleri* per liter, respectively; number in the square brackets correspond to the 95% confidence limits with the lower and upper values.
